# Informational stories: a complementary strategy for patients and caregivers with brain metastases

**DOI:** 10.3747/co.v16i3.397

**Published:** 2009-05

**Authors:** A.D. Chung, D. Ng, L. Wang, C. Garraway, A. Bezjak, J. Nyhof–Young, R.K.S. Wong

**Keywords:** Information needs, stories, narrative, patient education, brain metastases

## Abstract

**Objective:**

We compared the efficacy of a story-based writing style with that of a fact-based writing style for educational material on brain metastases.

**Methods:**

Identical informational content on four topics—radiation therapy, side effects, steroid tapering, and palliative care—was constructed into equivalent story-based and fact-based materials. The content and reader preference for style were evaluated using a questionnaire of 20 + 1 items. Cancer patients and caregivers were invited to evaluate the materials.

**Results:**

A total of 47 participants completed the questionnaire. The recorded preferences for facts, stories, or both were 42%, 7%, and 51% respectively (*p* = 0.0004). The fact-based materials were rated superior in providing factual information (for example, discussion of treatment, side effects) and selected general characteristics (clarity of information, for instance). A rating trend suggested that story-based materials were superior in describing “how it feels to have brain metastases” (21/40 fact-based vs. 26/43 story-based) and “how brain metastases affected a spouse” (17/41 fact-based vs. 21/47 story-based), and in being “sensitive to the frustrations of a patient with brain metastases” (25/40 fact-based vs. 30/44 story-based).

**Conclusions:**

Half the participants preferred to read both fact-based and story-based materials. A combined story-based and fact-based educational resource may be more effective in conveying sensitive information and should be further investigated.

## 1. INTRODUCTION

The challenges associated with cancer are often a source of emotional distress to cancer patients and their caregivers^1^. Brain metastasis, a common complication in cancer, presents in about 10%–30% of adult cancer patients^2^. Whether as a new diagnosis of advanced disease or a recurrence of previously treated malignancy, brain metastasis generally indicates reduced life expectancy, at a median of 3–6 months, given radiotherapy treatment^3^,^4^.

Most cancer patients wish to be fully informed about their illness^1^, but many continue to have a poor understanding of their situation^5^. Efficacious information exchange is key in helping patients to remain involved in their own care, to cope psychologically, and to develop realistic expectations^6^. Barriers to this information exchange include inadequate information presentation, fear and anxiety, and cognitive impairments from disease^1^,^5^,^7^. In particular, many patients facing the physical effects (for example, changes in physical or cognitive ability) and psychological effects (for example, preparing for end of life) of brain metastases may not be emotionally prepared to receive and retain information concerning their condition. This situation poses a unique challenge to clinicians in charge of their care.

Short written materials tend to be of aid to cancer patients and their caregivers in remembering information regarding their illness and in providing a resource to which they can refer multiple times^1^,^8^. Traditionally, such materials have been presented in a fact-based format^8^,^9^–^11^. However, story-based or narrative educational materials may be a more successful approach for presenting information to patients with brain metastases. Narrative educational materials have a number of plausible advantages over the traditional fact-based approach (addressed in detail in the Discussion section). Key features include the use of everyday language to which patients can better relate^12^, a cause-and-effect structure that unfolds as the story progresses^7^, the use of third-person fictional characters to describe experiences^7^, and a writing style that lends itself to creating a more empathetic approach^13^.

In the present research, we aimed to develop an evidenced-based educational resource useful for patients living with multiple brain metastases and for their caregivers, especially in cases in which life expectancy is anticipated to be short and palliative and end-of-life concerns are relevant issues. We therefore designed two sequential studies. First, health care professionals and cancer patients in general were invited to refine the informational content and writing style of our institutional resource. This paper reports the results of that first study. Later, patients with brain metastases and their caregivers will be invited to evaluate the final resource.

The goal of the initial study was to compare, for patients with brain metastases and for their caregivers, the value of using a more traditional fact-based approach to patient education (such as that found in conventional hospital education materials and consent forms) with a story-based narrative approach. The primary objective was to determine which presentation format patients and caregivers prefer. Secondary objectives were to explore potential differences between the preferences of patients and caregivers, and to identify potential areas for improvement in the resources.

## 2. PATIENTS AND METHODS

### 2.1 Theme Identification and Resource Development

Theme identification using a grounded theory approach was applied to transcribed interviews from two qualitative studies (39 interviews with 20 caregivers and 19 patients)^14^ designed to address the information needs of patients with brain metastases and of their caregivers. These key topics were identified:

- Future complications from brain metastases
- Radiation therapy
- Treatment side effects
- Steroid tapering
- Caregiver concerns
- Palliative care
- End-of-life concerns

Four of the foregoing topics were selected for use in the present study: radiation therapy, treatment side effects, steroid tapering, and palliative care. These topics were selected because existing fact-based educational resources on these topics were available for patients. For each of the four topics, separate fact-based resources (fact sheets) were created based on the written texts from existing pamphlets, booklets, and hospital consent forms^15^–^19^. Equivalent story-based resources consisting of fictional narratives 200–300 words in length were also written (by DN) for each of the four topics (Appendix a provides a representative sample).

### 2.2 Validation of the Resources

Health care professionals in cancer care were invited to use the study questionnaire to evaluate the new resources for both face validity and preference pattern. Modifications to the resources were made based on the feedback received.

### 2.3 The Questionnaire

A self-administered questionnaire was designed specifically to evaluate and compare the fact-based and story-based resources in terms of content, psychological effects, and preferences. The questionnaire consists of 20 statements exploring a respondent’s view of the factual content (“Factual information was clearly presented”) and emotional content (“The resource described how it feels to have brain metastases”) presented in the resources. Respondents used a 5-point categorical rating scale, with Strongly Disagree and Strongly Agree as the anchors, to score each statement. A final question inquired into the respondent’s resource preference (for stories, fact sheets, or both), and two open-ended questions inquired into why the respondent preferred a particular resource and allowed the respondent to include additional comments or suggestions for improvement.

### 2.4 Eligibility Criteria

For the current phase of our project, English-speaking cancer patients and their caregivers were eligible, although patients with brain metastases were specifically excluded. This strategy allowed us to obtain responses from individuals whose information needs we expected to be similar in style to those of patients and caregivers with brain metastases, but who would not be immediately confronted with the psychological stresses associated with a diagnosis of brain metastases.

### 2.5 Study Intervention

After informed consent was given, participants were provided with a study package. Study packages contained a consent form, a collection form for demographic information, the four stories, the four fact sheets, and the questionnaire. To eliminate potential order bias, packages labelled “A” were ordered with the stories first, packages labelled “B” were ordered with the fact sheets first, and similar numbers of both packages were distributed.

### 2.6 Statistical Considerations

A sample size of 37 evaluable questionnaires was planned to provide an 81% power to detect a difference of 0.2 using a one-sided binomial test. Results from the questionnaire were used in three ways. First, the proportion of participants expressing a preference for the stories, fact sheets, or both was used to determine the overall value of each presentation style. Second, overall and individual item scores were compared between the stories and the fact sheets for patients and for caregivers. Third, we explored whether any differences in response pattern emerged between the patients and the caregivers. A paired *t*-test was used for an overall comparison of fact sheets with narrative stories: mean scores were log-transformed, and a back-transformed mean difference was used. Individual items were compared using the Wilcoxon signed rank test. A non-paired *t*-test was performed to compare patient and caregiver responses. Because the item “would cause emotional distress for a patient” was a negative statement, the scale was reversed for that item in all statistical computations. Data from open-ended questions were transcribed by the primary investigator and analyzed qualitatively.

## 3. RESULTS

### 3.1 Validity of the Fact-based and Story-based Resources

The educational materials were evaluated for face validity by 11 health care professionals: 3 physicians, 6 oncology nurses, 1 radiation therapist, and 1 health educator. Based on the feedback received, modifications to the original versions of the resources were made (for example, description of side effects).

### 3.2 Patient and Caregiver Characteristics

Accrual for the study occurred between November 2004 and May 2007. Because of investigator interest and availability of summer-student researchers, most of the accrual occurred during the summer months from a gastrointestinal malignancy clinic. Questionnaires were completed by 47 respondents: 26 patients and 21 caregivers. Table i presents the characteristics of the respondents. Caregivers had a higher degree of education and were younger (median age 51) than were patient participants. Although most questionnaires had missing data, any given item on the questionnaire received at least 40 responses.

### 3.3 Preference for Fact-based or Story-based Resources

**FIGURE 1:**
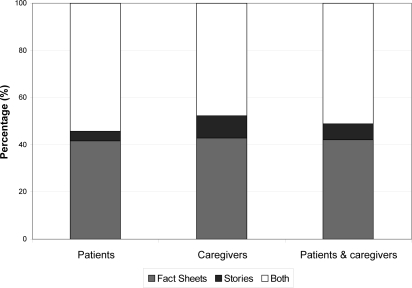
Preference of patients and caregivers for presentation styles.

Just over half the respondents [51% (23/45)] preferred to have both fact- and story-based resources. Another large proportion [42% (19/45)] of the respondents preferred the fact-based materials alone; just 7% (3/45) preferred the stories alone (*p* = 0.0004; Figure 1). Overall, respondents tended to score the fact sheets more favourably than the stories (*p* = 0.03).

Characteristics of, and potential differences in, the two resources were explored by examining individual items on the questionnaire. Statements on the fact sheets that scored below a 60% endorsement included “described how it feels to have brain metastases” and “described effect on spouse/partner/caregiver.” Statements on the stories that scored below a 60% endorsement included “helped understand brain metastases,” “offered information on planning for the future,” “described effect on spouse/partner/caregiver,” and “would comfort a patient.”

Respondents generally favoured facts over stories, with the difference being statistically significant for 10 of the 20 items. The fact sheets scored better in providing factual information (for example, discussed treatment, side effects, palliative care) and in some general characteristics (for example, information clearly presented, taught something new, explained new concepts well).

A trend in the responses suggested that stories better addressed “how it feels to have brain metastases” (21/40 fact-based vs. 26/43 story-based), better described “how brain metastases affect a spouse/partner/caregiver” (17/41 fact-based vs. 21/47 story-based), and were “sensitive to the frustrations of a patient with brain metastases” (25/40 fact-based vs. 30/44 story-based). Notably, concerns were also expressed that stories were more likely to “cause emotional distress for a patient with brain metastases” (12/43 fact-based vs. 32/44 story-based, Table ii).

The characteristics of each resource were further examined using the comments elicited by the open-ended questions about the stories and fact sheets. The comments indicated that respondents felt the stories were more “engaging to read,” so that the information in them might be better retained. Stories also had more of a “human touch” and “provided real-life scenarios that patients could relate to.” On the other hand, the fact sheets were seen as “more objective” and good for “referring back to.” Respondents provided suggestions for improving the resources, including a need to include a clear explanation of brain metastases, to address caregiver burden and distress, to refine content details, and to incorporate multicultural aspects.

### 3.4 Patients Compared with Caregivers

Overall, patients gave a higher score to the fact sheets than did the caregivers (4.29 vs. 4.01, *p* = 0.05). There was no significant difference between patients and caregivers with regard to the scoring of the story-based resources (Table iii).

## 4. DISCUSSION

Half the respondents expressed a preference to read both the story-based and the fact-based resources to address the information needs of brain metastases patients and their caregivers, a proportion that was similar to the proportion of respondents who preferred facts only. There is some suggestion that stories may be more effective in describing how it feels to have brain metastases, more sensitive to the frustrations of brain metastases patients, and better at describing the effect of the illness on a caregiver.

A diagnosis of brain metastases brings the fear of losing mental competency or of reaching the end of life itself. Although the facts regarding an advanced cancer are difficult to accept, patients and caregivers still desire information^14^,^20^. However, the information needs of patients and caregivers vary. Patients generally focus on current information needs; caregivers want more information about the future (for example, life expectancy and anticipated symptoms)^14^. The responsibility of health care providers and patient educators is to present the information in a sensitive manner that equips patients and caregivers alike with the necessary knowledge and that realigns hope against realistic expectations to facilitate successful coping with the diagnosis^14^.

Traditionally, written patient information has been presented in pamphlets, booklets, and brochures that use a fact-based approach. The concise and rational manner in which information is presented offers a high degree of clarity, thus making it a powerful teaching tool. Verghese *et al.* describe two aspects of illness, a physical deficit and a spiritual violation^21^. Although factual information is essential in the planning for and treatment of the physical deficit, the weakness of a fact-based resource is that it does not address the social, emotional, and motivational influences of illness. Most resources are designed to educate, but not to counsel^1^, despite the patient’s emotional need for a discussion of illness that crosses beyond the language of academic and professional analyses^22^. Thus, there is a need for more psychologically sensitive approaches to the design of educational resources.

A story-based format is by definition descriptive, inviting the reader to participate in another world in which their imagination allows them to see, feel, and hear what it is like to be there^22^. This approach is currently under development and has been applied, with encouraging results, in other emotionally charged issues such as testicular cancer^23^. A story-based approach has a number of likely advantages that have been explored in various contexts. Stories allow new concepts to be presented in language that is more familiar, bridging the gap between medical knowledge (which is possibly intimidating) and everyday experience, an approach that may be particularly useful in the presentation of new information^12^. The cause-and-effect structure of stories may permit better recollection of relevant information: that is, if one particularly memorable point of the story is remembered, the rest will follow; whereas with a fact-based format, each point is discrete^7^. The use of fictional characters in stories may be less threatening, allowing patients and caregivers to explore the issues of illness and mortality when they may be hesitant to do so themselves^7^. If patients and caregivers are able to relate to the characters, then those characters may even serve as models of action and help the patients or caregivers to feel less alone in their struggles^7^. A patient is less likely to contest a story than facts, making it easier for the patient to accept the reality of the situation and any intervention^7^. Stories use a writing style that lends itself better to the creation of empathy, thus addressing the emotional aspects of the situation for the patient or caregiver. Through the rich context of human actions, intentions, and experiences^24^, stories offer a window into an unfamiliar world^25^, allowing for a smoother transition from an unfamiliar situation to a familiar one. There are, however, limitations to a story-based approach. The information is embedded in a narrative and thus requires interpretation. Also, the emotional underpinning of the story-based format may carry a higher risk of causing psychological distress for some, although fiction may be a healthy context in which to explore those emotions^7^.

Because any patient population comprises a diverse cohort of individuals, some learning styles may better suit certain individuals. “Experience learning theory” describes learning as a 4-step cycle: feeling, watching, thinking, and doing^26^. Learners tend to have a preferential method of learning corresponding to one of the stages of the cycle^26^. Therefore, by providing a context that can be visualized and imagined, stories may serve to better educate patients and caregivers who need to feel, watch, or act on a situation to understand it. On the other hand, facts may better serve to educate patients and caregivers who learn best by conceptualizing and thinking and who benefit from a more didactic approach. By investigating both story-based and fact-based resources, the learning opportunities available to patients and caregivers are expanded.

Additional work is clearly required to further explore how and under what circumstances a story-based approach is best employed and what the potential role for a story-based approach may be for patients with brain metastases. One area for this work is to discern whether different sets of facts or different stories should be compiled for different audiences. Research indicates that a narrative format may be appropriate for more empathic individuals and that stories are more effective when readers can identify with the characters^7^. The success of such resources may thus depend on how well tailored they are for the specific audience. It has been suggested that individuals are most open to narratives immediately following a surprise or expectation failure^27^, and thus stories may be most appropriate soon after diagnosis^7^. There may be benefit in presenting information in small increments to allow for adjustment to diagnosis and treatment^8^, thus clearly marked signposts for certain types of information within the resource can possibly increase its usefulness. The combined strategy of stories supplemented by facts may provide the optimal approach and thus deserves further investigation.

## 5. CONCLUSIONS

A combination story-based and fact-based approach may be best suited to meeting the information needs of patients living with brain metastases and of their caregivers. This approach requires further investigation.

Fulfilling the information needs of patients with a poor prognosis, such as presented by multiple brain metastases, is challenging and difficult to do well. It is nonetheless important, because it can facilitate informed treatment decision-making, better symptom management, and good planning for the future. Currently, few resources are available that specifically meet these information needs. The use of a combined story-based and fact-based writing style may provide the optimal approach for constructing written materials. Studies formally addressing the effects of such a resource are needed and are currently ongoing.

## Figures and Tables

**TABLE I:** Characteristics of patients and caregivers at the time of study recruitment

*Characteristic*	*Patients*	*Caregivers*
Men:women (*n*)	18:8	11:10
Median age (years)	65	51
Highest level of education (%)		
University/professional	31	67
High school	50	29
Elementary school	19	5
First language (%)		
English	92	76
Non-English	8	24
Primary site (%)		
Esophageal	31	29
Colorectal	31	10
Other	38	62

**TABLE II:** Proportion of patients or caregivers (*n* = 47) assigning positive scores (≥4^a^) for individual items

*Question*	*Stories*	*Facts*	p *Value*^b^
Info clearly presented	39/47	46/47	0.0001
Taught me something new	34/46	43/47	0.0003
I would give this to someone with brain metastases	36/46	45/47	0.003
Helped understand brain metastases	27/47	35/46	0.006
The overall content was well organized	41/47	44/47	0.02
Patients will find this useful	37/46	43/46	0.02
Explained new concepts well	30/45	41/46	0.02
Discussed palliative care	35/42	40/42	0.04
Discussed treatment	33/42	38/41	0.04
Discussed treatment side effects	37/42	41/42	0.05
Offered information on planning for the future	17/42	25/41	0.07
Info relevant to brain metastases	37/46	40/43	0.13
The length of the resource was appropriate	41/46	42/46	0.16
Described how it feels to have brain metastases	26/43^c^	21/40	0.25
Described effect on spouse/partner/caregiver	21/44^c^	17/41	0.27
Would comfort a patient	24/44	28/43	0.29
Sensitive to the frustrations of patient	30/44 c	25/40	0.57
Would cause emotional distress for a patient^d^	14/44	12/43	0.92
Provided info on caring for patient	29/42	29/41	0.94
Easy to understand	46/47	47/47	1.00

^a^ 1 = Strongly Disagree; 5 = Strongly Agree.

^b^ Calculated using a Wilcoxon signed rank test applied to scores on a 5-point rating scale.

^c^ Demonstrate a trend suggesting that stories are more effective than facts.

^d^ Negative statement.

**TABLE III:** Comparison between caregivers and patients

*Questions*	*Group*	*Mean*^a^	p *Value*^b^
Fact	Caregivers	4.01	0.05
	Patients	4.29	
Story	Caregivers	3.87	0.55
	Patients	4.01	

^a^ Score for the negative statement was reversed before the mean was calculated.

^b^ Calculated using a *t*-test applied to scores on a 5-point rating scale.

## APPENDIX A

### SAMPLE FACT- AND STORY-BASED RESOURCES

#### Radiation Therapy—Story-based

Amelia woke up with butterflies in the pit of her stomach. After undergoing a mastectomy 2 years ago for breast cancer, she had recovered well and hoped that her cancer was gone for good. This new diagnosis of brain metastases had hit her like a ton of bricks. She wondered why this was happening to her again. As she dressed for her appointment at Princess Margaret Hospital, she wondered what radiation therapy would be like. Would she feel anything? Would her brain be radioactive after? What if she felt claustrophobic?

In the waiting room, Amelia felt her husband’s hand squeeze hers reassuringly as she tried to stay calm. Her husband, Wayne, would be allowed into the room with her, but would have to leave when they turned on the radiation. Knowing that he would be able to watch her on a screen just outside the room was comforting, though.

Once inside the room, the radiation therapists positioned her on the bed. They then placed a piece of tape from the edge of the treatment couch across her forehead to remind her to keep her head still during her treatment. She felt trapped and slightly afraid. As the CD she had brought started to play, Sarah McLachlan’s soothing voice helped to ease her anxiety.

Suddenly, the radiation therapists were turning off the lights and leaving the room, and Amelia realized that the moment had come. She slowly took three breaths and the light came back on. She hadn’t felt a thing! “Wow, this wasn’t bad at all,” she thought with relief.

Twenty minutes later, she was back in the doctor’s office with Wayne and discussing potential side effects. No longer afraid, Amelia knew that 4 more rounds of this wouldn’t be too difficult. Plus, if it just managed to shrink some of those tumours ...

#### Radiation Therapy—Fact-based

Radiation therapy uses X-rays, gamma rays, or electrons to treat cancer. For brain metastases, radiation therapy is given externally by machines and is called external-beam radiation.

Radiation treatment is like having an X-ray taken. You won’t feel any pain or sensation and you will not become or remain radioactive. Try to stay relaxed, breathe normally, and remain in the position that the Radiation Therapist puts you in. You may hear clicking or whirring noises while the treatment machine is on. When the treatment is being given, the Radiation Therapists will not stay in the room with you, but they will be able to see you at all times on a television screen.

The length of your daily treatment depends on the radiation dosage to be given and the machine being used. The treatment beam is only on for a short period of time. If you would like, you can bring your own music on tapes or CD, which can be played during your treatment.
